# Removal of cadmium from aqueous solutions using industrial coal fly ash-nZVI

**DOI:** 10.1098/rsos.171051

**Published:** 2018-02-14

**Authors:** Lixia Ma, Qi Wei, Yueqin Chen, Qiuyang Song, Conghui Sun, Zhiqiang Wang, Guanghong Wu

**Affiliations:** 1 Tianjin Key Laboratory of Water Resources and Environment, Tianjin Normal University, Tianjin 300387, People's Republic of China; 2 College of Urban and Environmental Sciences, Tianjin Normal University, Tianjin 300387, People's Republic of China

**Keywords:** nano zerovalent iron, coal fly ash, cadmium (II), adsorption, wastewater

## Abstract

Batch experiments were conducted to test the effects of various solution properties, such as pH, temperature, initial concentration and anoxic and aerobic atmosphere, on Cd removal by nanoscale zerovalent iron (nZVI) supported on industrial coal fly ash. Cd (II) could be removed by adsorption on fly ash-nZVI in a very short time (5 min) with high removal rates (greater than 99.9%) over a wide range of concentration (5–100 mg l^−1^). Cd (II) was physically adsorbed on the surface of fly ash-nZVI. The preparation of fly ash-nZVI can incorporate the use of waste media, making the overall adsorbent more removal efficient and low cost.

## 1. Introduction

Heavy metal pollution of wastewater is one of the most important environmental problems throughout the world. Cd and Cd compounds, which were classified as potent carcinogens by the International Agency for Research on Cancer in 1983, can cause damage to the lungs, kidneys, liver and reproductive organs. Cd is ranked as the seventh most important hazardous substance in the USA [1] and has resulted in serious contamination of soils, water, sediments and organisms in China. Cd is commonly found in effluents from a wide range of industries, such as non-ferrous metals production, electroplating, and manufacturing of electronic products. Consequently, great attention is required for the removal of Cd from wastewater before it reaches water bodies. In recent years, various methods (chemical precipitation, ion exchange, adsorption, membrane filtration and electrochemical treatment) for Cd removal from wastewater have been studied to meet the increasingly stringent environmental regulations [2–4]. Adsorption is a recognized method for heavy metal removal from low-concentration wastewater, which has been developed as a simple, efficient and cost-effective treatment for removing heavy metals. A number of adsorbents have been used for Cd removal including activated carbon adsorbents, zeolites, nano zerovalent iron (nZVI), carbon nanotube adsorbents, low-cost adsorbents and bioadsorbents. Adsorption by low-cost adsorbents, as an alternative to activated carbon, is recognized as an effective and economical method for low-concentration heavy metal wastewater treatment. Industrial coal fly ash is a low-cost and better adsorbent due to its abundance and environmental friendliness. The primary compositions of industrial coal fly ash are silica (SiO_2_) and alumina (Al_2_O_3)_. Fly ash and Bayer residue can successfully adsorb TOC, nutrients and Cu [4]. Sorbents synthesized from coal fly ash and geopolymer are efficient for lead removal [5]. However, coal fly ash removes heavy metal ions with low efficiency. Zerovalent iron nano particles have been investigated as a new material for the treatment of contaminated water. The equilibrium cadmium adsorption with 0.5 g l^−1^ nZVI can reach up to 213 mg g^−1^ at 285 K and 225 mg g^−1^ at 333 K [6]. Li *et al*. reported that the average Cu (II) removal efficiency was greater than 96% with 0.20 g l^−1^ nZVI and an agitation time of 100 min [7]. Nevertheless, the application of nZVI presents some limitations such as rapid oxidation, rapid aqueous aggregation, production costs and recovery of the nano materials (with associated contaminants) [8,9]. There is a need for more detailed and systematic studies on the removal mechanism of contaminants and technical improvements in nZVI synthesis [9]. In the present study, nanoscale zerovalent iron supported on fly ash (fly ash-nZVI) is prepared and tested for its ability to remove Cd from aqueous solutions.

The main objectives of the present study are to (i) determine the effect of fly ash-nZVI on heavy metal remediation in wastewater, (ii) characterize fly ash-nZVI and its reaction products using microscopy and (iii) investigate the different environmental factors on Cd remediation and the removal mechanism using spectroscopy.

## 2. Material and methods

### 2.1. Chemicals

Industrial coal fly ash was provided by Dagang Coal-fired Power Plant (Tianjin, China). The primary compositions of industrial coal fly ash are 62.1% SiO_2_, 25.8% Al_2_O_3_, 1.2% Fe_2_O_3_, 4.16% CaO, 1.02% MgO and 3.02% loss on ignition (LOI) by weight. CdCl_2_ solution (1 g l^−1^, GBW08612) was provided by National Center for Certified Reference Materials (Beijing, China). Iron (III) chloride hexahydrate (FeCl_3 _· 6H_2_O) and sodium borohydride (NaBH_4_) were purchased from Tianjin Fuchen Chemicals Reagent Factory (Tianjin, China). All chemicals were of analytical grade purity.

### 2.2. Preparation of fly ash-nZVI

Fly ash-nZVI was prepared using a conventional liquid-phase method via the reduction of ferric ion (FeCl_3 _. 6H_2_O) by NaBH_4_ with industrial coal fly ash as the support material. A detailed preparation of nZVI was provided in [10]. Briefly, a 1.0 M FeCl_3_ aqueous solution was added dropwise into industrial coal fly ash at ambient temperature with magnetic stirring. Next, a 1.6 M NaBH_4_ aqueous solution was added dropwise into the suspension with magnetic stirring. The wet Fe was precipitated on the surface of industrial coal fly ash. Coal fly ash-nZVI was stored in brown bottles and coated with ethanol for protecting from oxidation.

### 2.3. Batch experiments

The effects of different experimental conditions on the removal efficiency and kinetics of Cd were studied in the procedure, as described below. Different dosages of fly ash-nZVI were added into the wastewater with different initial Cd concentrations at different temperatures (288–308 K) with magnetic stirring for more than 1 h. Then wastewater was extracted in 5 min and then every 10 min using a 10 ml dispensable syringe and filtered through a 0.22 µm filter for further analysis. All experiments were performed in triplicate.

### 2.4. Characterization and analytical methods

The morphological analyses of coal fly ash and fly ash-nZVI were performed using a scanning electron microscope (SEM, S4800, Hitachi). The morphological analyses of coal fly ash and fly ash-nZVI was also performed using a transmission electron microscope (TEM, Tecnai G2 F20, FEI). X-ray photoelectron spectroscopy (XPS, 250 Xi, Thermo) analysis was conducted on coal fly ash and fly ash-nZVI before and after reacting with Cd. The concentrations of Cd in aqueous samples were determined using inductively coupled plasma optical emission spectroscopy (ICP–AES, Optima 7300 V, PE), or inductively coupled plasma mass spectrometry (ICP–MS, Elan 9000, PE) for more sensitive metal determination.

### 2.5. Calculation of removal rate and adsorption capacity

The removal rate (*η*, %), the amount of Cd (II) adsorbed per unit mass of adsorbent at time *t* (*q_t_*, mg Cd per g nZVI) and the amount of Cd (II) adsorbed per unit mass of adsorbent at equilibrium (*q*_e_) were calculated from the following equations [3]:

2.1 $$\begin{array}{r} \eta =\frac{C_{0}-C_{e}}{C_{0}}\times 100\%, \end{array}$$

2.2 $$\begin{array}{r} q_{t}=V\times \frac{C_{0}-C_{t}}{m_{s}} \end{array}$$

2.3 $$\begin{array}{r} \text{and}\;\;\;\;q_{e}=V\times \frac{C_{0}-C_{e}}{m_{s}}, \end{array}$$

where *C*_0_ and *C_e_* (mg l^−1^) are the initial and the final concentrations of Cd (II) in the solution, respectively, and *C_t_* (mg l^−1^) is the concentration of Cd (II) at time *t*. *V* is the volume of the solution (l) and *m_s_* is the mass of dry adsorbent (nZVI) used (g).

### 2.6. Kinetic models

The sorption kinetics of Cd (II) was tested using pseudo-second-order sorption equations. The pseudo-second-order equation can be written as [3]:

2.4 $$\frac{t}{q_{t}}=\frac{1}{k_{2}q_{e}^{2}}+\frac{t}{q_{e}},$$

where *k*_2_ (g(mg min)^−1^) is the rate constant of the pseudo-second-order sorption.

## 3. Results and discussion

### 3.1. Surface analysis of fly ash-nZVI

**Figure 1.**
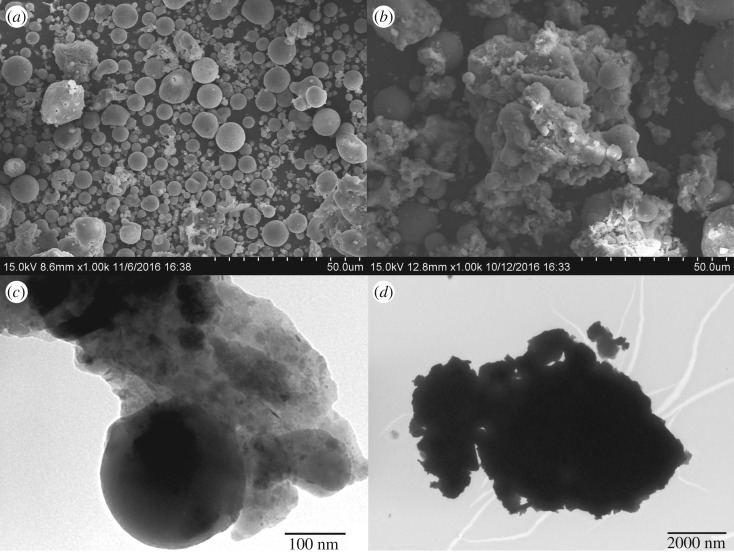
Particle size distribution of (*a*) fly ash particles; (*b*) fly ash-nZVI particles after Cd adsorption; (*c*) TEM image of fly ash-nZVI and (*d*) TEM image of fly ash-nZVI after Cd adsorption.

The adsorbent shape and size impact the adsorption capacity of the adsorbent. SEM and TEM measurements were conducted on fly ash, fly ash-nZVI, and fly ash-nZVI after Cd adsorption (figure 1). SEM images of fly ash particles indicated that the material is composed of individual, spherical particles that form aggregates (figure 1*a*). The size of the spherical particle was approximately 0.5–10 µm. The main compositions of industrial coal fly ash were 62.1% SiO_2_ (the spherical particle) and 25.8% Al_2_O_3_ (the floccus) by weight. This small particle size provides a larger surface area for contaminant adsorption. SEM analysis was conducted to evaluate the adsorption of Cd (II) on fly ash-nZVI particles (figure 1*b*), which showed that spherical particles with flocculent were present as well as the presence of larger flocs, possibly from aluminium oxide formation. TEM analysis showed that nZVI particles, which were nearly spherical in shape and uniform in size with a mean diameter of 80–120 nm, were distributed dispersedly on the surface of coal fly ash (figure 1*c*). The TEM image of coal fly ash-nZVI after Cd adsorption (figure 1*d*) showed that the spherical particles were not present but also indicated the presence of larger flocs. Many changes took place due to the combined effects of co-precipitation, adsorption and other complex cooperation between coal fly ash-nZVI and wastewater.

### 3.2. Effect of pH

**Figure 2.**
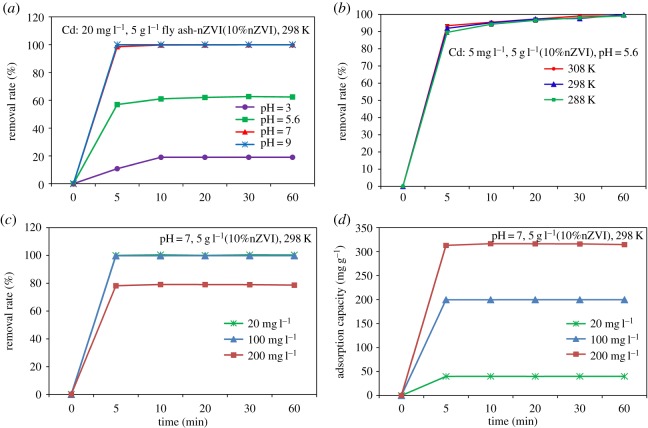
Kinetics of Cd (II) adsorption: (*a*) the effect of pH on removal rate, (*b*) the effect of temperature on removal rate, (*c*) the effect of initial concentration on removal rate and (*d*) the effect of initial concentration on adsorption capacity.

Adsorption experiments of Cd (II) on fly ash-nZVI were performed at different pH values (3.0–9.0), three different temperatures (288–308 K), different initial Cd (II) concentrations (5–200 mg l^−1^) and oxygen concentrations in order to investigate their influence. The effect of pH (3.0–9.0) on the adsorption of Cd (II) (20 mg l^−1^) on fly ash-nZVI was first investigated (figure 2*a*). It could be seen that the removal rates of Cd (II) increased with an increase in the initial pH (3.0) and the removal rates were invariant above pH 7.0. Below pH 8.0 Cd is present as Cd (II). And yet above pH 8.0, various Cd hydroxide species (i.e. CdOH^+^, Cd_2_(OH)^3+^, Cd(OH)_2_, Cd(OH)^3−^ and Cd(OH)_4_^2−^) start to form. Cd hydroxide species are stable colloids and are transported into the pores of fly ash particles. Consequently, Cd hydroxide precipitation might have contributed to its removal from solution at pH greater than 8.0. Cd (II), Fe^0^, FeO, Fe_2_O_3_ and other iron oxides (e.g. Fe(OH)_2_) are present in the aqueous solution and the interactions of Cd (II) and Fe (III) particularly, promote the formation of cadmium ferrite (CdFe_2_O_4_). The aggregation of fine particles of CdFe_2_O_4_ has two kinds of shapes, fibrous and granular in high-resolution TEM [11]. Co-precipitation with coal fly ash-nZVI is among the main mechanisms responsible for Cd removal. Boparai *et al*. reported that Cd removal increased with solution pH and reached a maximum at pH 8.0 [12]. In the present study, the removal rates were all more than 99.9% in the pH range of 7–9. Initially, the adsorption was fast (i.e. first 5 min), and finally approached equilibrium. The initial fast adsorption might be due to the large amount of adsorptive sites available. The effect of pH on Cd sorption is also related to the changes in the surface charge and can be explained in terms of point of zero charge. At pH above 7.9, the nZVI surface acquires a net negative charge making the surface electrostatically favourable for higher adsorption of Cd (II) [12]. Most of the Cd (II) was adsorbed at pH 7 (figure 2). The results indicate that the non-specific sorption due to electrostatic attractions between Cd (II) and nZVI surface is unlikely to be the major mechanism for Cd (II) adsorption and Cd (II) ions are adsorbed on the nZVI surface by specific sorption rather than non-specific sorption.

### 3.3. Effect of temperature

Temperature is an important parameter that can influence the sorption process. The effect of temperature on the adsorption of cadmium by nano zerovalent iron was studied from 288 to 308 K at initial Cd concentration of 5 mg l^−1^ and nZVI dosage = 0.5 g l^−1^. The results showed that the adsorption rate of Cd (II) ions was very fast initially, and approximately 99.9% of total Cd (II) was removed within 5 min. An increase in the temperature resulted in a slightly increased cadmium adsorption rate initially (5 min) (figure 2*b*). Nevertheless, the removal rate and equilibrium cadmium adsorption were relatively insensitive to temperature. The result was consistent with a previous study [12]. Higher removal rates (99.9%) were found even at low initial Cd (less than 5 mg l^−1^) and low pH value (pH = 5.6). Wang *et al*. reported that approximately 40.0% of total Cd (II) from aqueous solution by a new low-cost adsorbent-Bamboo charcoal [3]. Soto Hidalgo *et al*. exposed nZVI to 6 mg l^−1^ of Cd (II) and found that the nZVI remediation efficiency of cadmium ions was between 80% and 90% in aqueous media [11]. The results showed that the nZVI remediation efficiency of Cd can be improved when nZVI is coated by fly ash. Boparai *et al*. reported that an increase in the temperature resulted in an increased cadmium adsorption rate, and the adsorption of Cd (II) by nZVI was endothermic [12].

Previous research has investigated that Cd adsorption follows pseudo-second-order kinetics and the pseudo-second-order kinetic model assumes that one cadmium ion is absorbed onto two sorption sites on the nZVI surface [12]. The effect of temperature on the adsorption kinetics of Cd (II) was studied in a previous study and an expression of the pseudo-second-order rate based on the solid capacity was presented for the kinetics of sorption [12,13]. Kinetic parameters, including the second-order rate constant (*k*_2_), calculated equilibrium adsorption capacity for Cd (II) (*q*_e_), the initial adsorption rate (*h*) and regression coefficients (*r*^2^), were investigated in the present study (table 1). The results were in accordance with the previous studies [12,13].

**Table 1.** Pseudo-second-order kinetic parameters of Cd (II) removal under different temperatures.

temperature (K)	*q*_e_ (mg g^−1^)	*k*_2_ (g mg^−1^ min)	*h* (mg g^−1^ min)	*r^2^*
288	322	0.0138	1430.8	0.996
298	316	0.0349	3484.9	0.998
308	316	0.0654	6535.2	0.999

### 3.4. Effect of initial concentration

Experiments were performed to determine the effect of initial concentration of Cd (II) on the removal rate and adsorption capacity of fly ash-nZVI (figure 2*c*,*d*). Cadmium adsorption was significantly influenced by the initial concentration of cadmium in aqueous solutions. The removal rate of Cd decreased from 100% to 80% with an increase in the initial Cd (II) concentration from 20 mg l^−1^ and 100 to 200 mg l^−1^ (figure 2*c*). The adsorbed amounts were 40.1, 200.0 and 316.0 mg g^−1^ for 20, 100 and 200 mg l^−1^ Cd (II), respectively (figure 2*d*), indicating that the adsorption capacity to Cd (II) was raised with an increase in the initial concentration of Cd (II).

Gaseous nitrogen was not employed to achieve the anoxic atmosphere in the present study. However, the removal rate and adsorption capacity did not increase significantly compared to when gaseous nitrogen was employed to achieve an anoxic atmosphere, which may be because coal fly ash-nZVI is deposited in aqueous solution and adsorption occurs in aqueous solution. The results indicate that nZVI on coal fly ash was better protected against fast oxidation.

**Figure 3.**
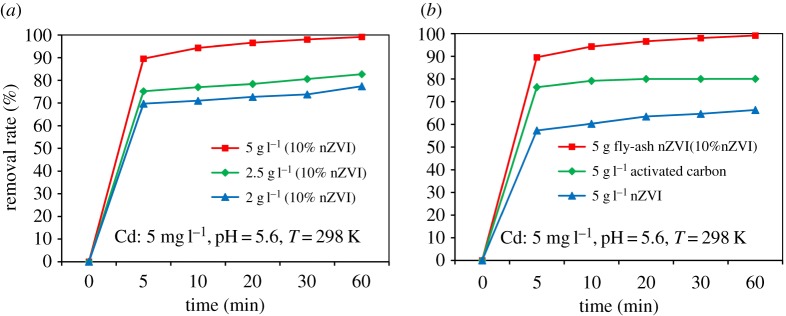
Comparison of Cd adsorption: (*a*) at different amounts of fly ash-nZVI, (*b*) on activated carbon, nZVI and coal fly ash-nZVI.

Cd (II) sorption at different amounts of coal fly ash-nZVI is shown in figure 3*a*. The removal rates increased progressively with the amount of fly ash-nZVI. The high removal rates might be due to the large amount of adsorptive sites available at 5 g l^−1^ coal fly ash-nZVI (10% nZVI); as the adsorption sites gradually increased, the removal rates became larger. Coal fly ash was found to successfully adsorb Cd (II) in the very low initial concentration (0.10–0.15 mg l^−1^) under alkaline condition (pH > 8.5). The Cd (II) adsorption performances of different adsorbents (activated carbon, nZVI and coal fly ash-nZVI) were also investigated in the present study. The removal rates of different types of adsorbents used for the removal of Cd (II) was compared (figure 3*b*) and it was found that fly ash-nZVI may be a highly efficient adsorbent for the removal of Cd (II) ions from wastewater.

### 3.5. Adsorption capacities of Cd

Numerous studies have investigated Cd (II) adsorption by various adsorbents, and the adsorption capacity (mg Cd per g nZVI) of some adsorbents used for the removal of Cd (II) was compared (table 2). Bagasse fly ash and bamboo charcoal may be highly efficient low-cost adsorbents for the removal of Cd (II) ions from water, and the maximum Cd (II) adsorption capacities were 6.19 and 12.08 mg g^−1^, respectively [3,14]. Chitosan nanoscale zerovalent iron (CS–nZVI) and green synthesis of iron nanoparticles (Fe NPs) were used for the remediation of Cd (II) from aqueous solutions; the maximum Cd (II) adsorption capacity was 120 mg Cd (II)/g CS–NZVI [15]; the Cd (II) removal capacity of Fe NPs synthesized by *Ilex latifolia* leaf extracts was 108 mg Cd (II)/g Fe [13]. From the results of control experiments, it can be seen that a higher adsorption capacity is mainly due to the contribution of nZVI particles. Fly ash-nZVI has the highest adsorption capacity, which may be explained by the fact that nanoscale iron particles were better dispersed on fly ash than bulk nano iron. Soto Hidalgo *et al*. reported that from high-resolution TEM images, nanofibre formation of a mixture of Fe^0^, oxyhydroxides and oxides of iron occurred after interacting with cadmium ions, possibly forming CdFe_2_O_4_ and FeOOH shell and other iron oxides in nZVI, which could enhance Cd (II) removal [11]. The oxidation of nZVI on coal fly ash did not occur for the adsorption of Cd (II). Maybe the formation of CdFe_2_O_4_ occurred after the adsorption of Cd (II). The higher adsorption capacity for Cd (II) in the present study may be due to the complex cooperation between fly ash and nZVI, which could cause a change of the initial structure of nZVI to nanofibres due to the possible formation of CdFe_2_O_4_ as a waste product.

**Table 2.** Comparison of adsorption capacities of Cd (II) ions with different adsorbents.

adsorbent	initial Cd (mg l^−1^)	time (min)	removal rate (%)	adsorption capacity (mg g^−1^)	references
bagasse fly ash	14	60	90	1.20	[14]
coconut charcoal	100	300	62.8	3.14	[15]
bamboo charcoal	100	360	>40	18.20	[3]
Fe NPs	10	60	41.7	2.09	[16]
CS-nZVI	100	60	>65	130	[13]
fly ash-nZVI	100	5	99.9	200	in the present study

### 3.6. Removal mechanisms of Cd

**Figure 4.**
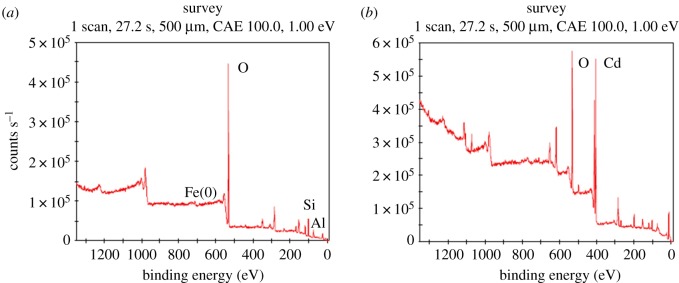
Typical wide-scan XPS spectra of the fly ash before (*a*) and after (*b*) Cd (II) adsorption. Initial concentration of Cd (II): 100 mg l^−1^, fly ash-nZVI: 1 g (10% nZVI), pH: 7.0, temperature: 298 K, time: 60 min.

The results of XPS characterization of fly ash-nZVI before and after Cd (II) adsorption are shown in figure 4. The XPS spectrum of fresh fly ash indicated the presence of O, Si, Al and Fe in the structure (figure 4*a*) but did not show the characteristic signal of Cd (II) ions on the surface of fresh fly ash. A new peak at the binding energy of 405 eV appeared after 1 h exposure of fly ash-nZVI to Cd (II) (figure 4*b*), which was assigned to the photoelectron peak of Cd. The results of XPS characterization showed that before Cd (II) adsorption, the mass ratio of SiO_2_, Al_2_O_3_, Fe_2_O_3_ and Cd was 62.1%, 25.8%, 1.2% and 0%, respectively. After Cd (II) adsorption with initial Cd concentrations of 100 and 200 mg l^−1^, the mass ratio of Cd to Fe increased from 0 to 6.4 and 8.0, respectively. The phenomena indicated the uptake of Cd on the surface of fly ash-nZVI.

**Figure 5.**
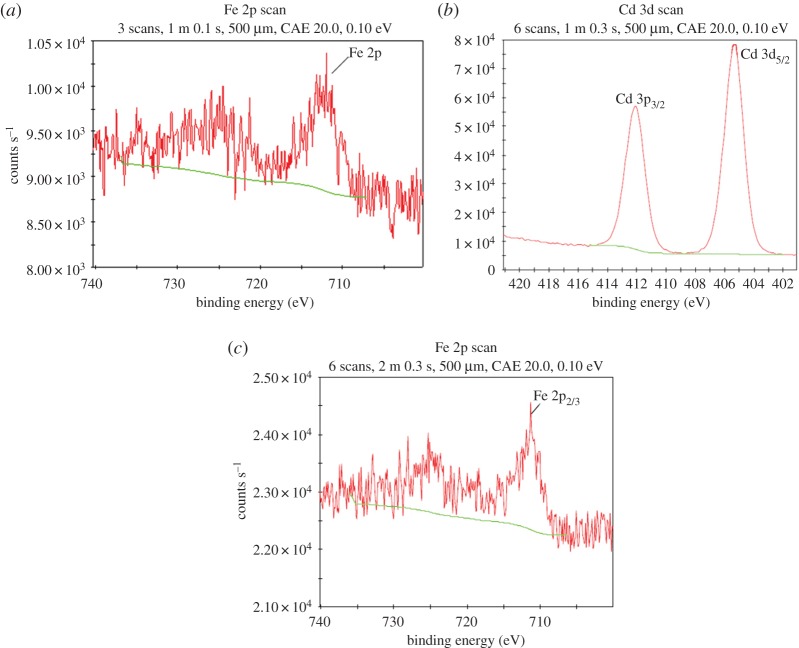
High-resolution XPS survey of (*a*) Fe 2p before adsorption, (*b*) Cd 3d and (*c*) Fe 2p after adsorption. Initial concentration of Cd (II): 100 mg l^−1^, fly ash-nZVI: 1 g l^−1^ (10% nZVI), pH: 7.0, temperature: 298 K, time: 60 min.

Detailed XPS surveys on the region of Cd 3d and Fe 2p are shown in figure 5. The peak of Fe 2p before adsorption was observed in high-resolution XPS survey (figure 5*a*), and the photoelectron peak for Cd after adsorption was centred at 405 and 412 eV (figure 5*b*). The peak positions for the different Cd 3d XPS features are relatively invariant and the 405 eV peak position associated with the Cd 3d_5/2_ level is in accordance with prior XPS-based work of Cd (II) in the literature [12,16]. Cd 3d_3/2_ and Cd 3d_5/2_ peaks were observed in the present study through high-resolution XPS, which is in accordance with the results in a previous study [11]. The results indicated that Cd (II) is captured within coal fly ash-nZVI by adsorption or surface complex with no apparent reduction on the nZVI surface. XPS analysis confirmed that Cd (II) was adsorbed onto the fly ash-nZVI particles and Cd (II) was physically adsorbed without being oxidized on the surface of fly ash-nZVI. Owing to a high removal rate (more than 99.9%), the reaction went on for only 5 min in the present study. The high-resolution Fe 2p spectra indicated the presence of different valence of iron (figure 5*c*). A prominent peak at 711 eV corresponding to the binding energy of Fe 2p_3/2_ indicated the presence of Fe^3+^ in the coal fly ash-nZVI particles after Cd adsorption. However, no peak at 724 eV was found, which may be present as Fe 2p_1/2_, indicated the absence of Fe^2+^ in the fly ash-nZVI particles. The findings indicated that the oxidation of nZVI on coal fly ash occurred after the adsorption of Cd (II). The results of the present study showed that Cd was removed as Cd (II) ions by adsorption on the fly ash-nZVI surface, and fly ash-nZVI was an efficient material for the treatment of Cd.

The compositions of coal fly ash are usually dependent on the raw coal. It should be noted that the variable composition of coal fly ash may influence the capacity of stabilization of cadmium. The components of coal fly ash are SiO_2_, Al_2_O_3_ and Fe_2_O_3_, CaO, K_2_O, Na_2_O, MgO and LOI. Alkalinity may increase with more CaO, K_2_O, Na_2_O and MgO contained in coal fly ash-nZVI. The experimental results indicated that the adsorption capacity increased with the increase of pH values accordingly. The capacity of Cd stabilization increased with the increase of the content of alkaline earth metal oxide in coal fly ash. The results suggested that coal fly ash-nZVI, which contained high CaO, K_2_O, Na_2_O and MgO, was much more efficient than other coal fly ash-nZVI, and Cd(II) could be easily recovered from wastewater using this coal fly ash-nZVI.

The coal fly ash-nZVI process presented was designed for electroplating wastewater treatment. A pilot test was conducted using 2.0 kg coal fly ash-nZVI to treat a total of 5000 l wastewater containing a high level of Cd (II). Low (0.1 mg l^−1^) and high (100 mg l^−1^) concentrations of Cd (II), Pb (II), Cu (II) and Zn (II) can be easily adsorbed on coal fly ash-nZVI due to the combined effects of co-precipitation and adsorption. Coal fly ash-nZVI was chemically stable in its chemical property as Cd concentration was below 0.01 mg l^−1^ all along and the concentration of released iron ion was below 0.02 mg l^−1^. After remediation coal fly ash-nZVI contained heavy metals, which belong to hazardous wastes in China, and should be disposed by a company that specializes in the disposal of hazardous wastes.

## 4. Conclusion

Nano zerovalent iron supported on industrial coal fly ash was successfully prepared. Coal fly ash-nZVI was an effective adsorbent to capture low-concentration Cd (less than 100 mg l^−1^) in wastewater. Coal fly ash could promote the formation of flocculent and the separation of Cd (II), even at low concentrations. XPS analysis confirmed that Cd (II) was adsorbed onto the fly ash-nZVI particles. nZVI on industrial coal fly ash can better protect against aggregation and oxidation, and Cd (II) could be effectively removed from contaminated water sources by adsorption on fly ash-nZVI in a very short time at a lower pH value. It is significant to note that the recovery of fly ash-nZVI (with associated contaminants) is practical and feasible. The results suggest that fly ash-nZVI can be effectively used for the removal of cadmium from contaminated water sources.

## Data accessibility

All of the data in this investigation have been reported in the paper and are freely available.
